# Construction and validation of a nomogram model for predicting response to initial radioiodine therapy in differentiated thyroid cancer patients using serum cytokines combined with serum thyroglobulin

**DOI:** 10.3389/fendo.2026.1803268

**Published:** 2026-06-02

**Authors:** Guohua Qin, AiQiang Dong, Ning Wang, Xinfeng Liu, Yingying Zhang, Guoqiang Wang, Chenghui Lu, Jiao Li, Na Han, ZengHua Wang, Zengmei Si, Fengqi Li, Rui Wang, Congcong Wang, Xufu Wang

**Affiliations:** 1 Department of Nuclear Medicine, The Affiliated Hospital of Qingdao University, Qingdao, Shandong, China; 2 Department of Nuclear Medicine, Anqiu People’s Hospital Affiliated with Shandong Second Medical University, Anqiu, Shandong, China; 3 Department of Disease Prevention and Control, Qingdao Women and Children’s Hospital, Qingdao Shandong, China; 4 Department of Thyroid Surgery, Qingdao Central Hospital, University of Health and Rehabilitation Sciences, Qingdao, China

**Keywords:** cytokines, differentiated thyroid cancer, prediction, radioactive iodine therapy, therapeutic response

## Abstract

**Aim:**

To investigate the predictive value of serum cytokines for treatment response to initial radioactive iodine therapy (RAI) in patients with intermediate- or high-risk differentiated thyroid cancer (DTC), and to develop a novel nomogram model for this purpose.

**Materials and methods:**

This study conducted a retrospective analysis of 429 patients with intermediate- or high-risk DTC who underwent initial RAI therapy across two medical institutions. The training cohort (n = 300) was derived from the Affiliated Hospital of Qingdao University, while the external validation cohort (n = 129) was recruited from Qingdao Central Hospital, University of Health and Rehabilitation Sciences. Treatment response to initial RAI was assessed based on follow-up data collected 6 to 12 months post-treatment. Patients were categorized into excellent response (ER) and non-excellent response (non-ER) groups in accordance with the 2025 American Thyroid Association (ATA) Response Evaluation System. Univariate and multivariate logistic regression analyses were performed within the training cohort to identify independent predictors of treatment response, followed by the development of a nomogram model. The model was subsequently subjected to external validation using calibration curves, decision curve analysis (DCA), and receiver operating characteristic (ROC) curve analysis.

**Results:**

In the training cohort, serum thyroglobulin (sTg) (OR = 0.938, P < 0.001) and serum interferon-alpha (IFN-α) (OR = 1.446, P < 0.001) were identified as independent risk factors for treatment response to initial RAI therapy in patients with intermediate- or high-risk DTC. The ROC curve of the nomogram model developed using the training cohort exhibited acceptable predictive performance. Calibration curves revealed a high degree of concordance between predicted probabilities and observed outcomes. DCA confirmed the potential clinical utility of the model. These findings were further corroborated in the external validation cohort.

**Conclusions:**

Based on systematic modeling and external validation, this study integrated sTg and IFN-α to develop and externally validate a novel nomogram for predicting treatment response to initial RAI therapy in patients with intermediate- or high-risk DTC, thereby offering evidence-based support for individualized risk stratification and follow-up planning.

## 1 Introduction

Thyroid cancer is one of the most common endocrine malignancies, and differentiated thyroid cancer (DTC) accounts for more than 90% of thyroid cancer cases (1, 2). The prognosis of DTC is relatively favorable, with recent research indicating that the 5-year overall survival rate of DTC is approximately 98% (3). According to the risk-adapted management framework of the 2025 version of the American Thyroid Association (ATA) guidelines, the use of postoperative radioiodine therapy (RAI) should be individualized based on the risk of recurrence, postoperative disease burden, imaging results, and biochemical indicators. For patients with intermediate- to high-risk DTC, RAI should be used to eliminate residual thyroid tissue or microscopic metastatic lesions and facilitate subsequent disease monitoring (3, 4). Thyroglobulin (Tg), which is a significant serum marker of thyroid cancer, is typically employed to evaluate the therapeutic effect of RAI in DTC patients (4–7). Recent studies have confirmed that the immune status of the body influences the therapeutic effect of initial RAI in intermediate- and high-risk DTC patients (8).

Cytokines, which are crucial regulatory factors of the human immune system, can modulate the tumor microenvironment and affect radiosensitivity (9–11). To date, relatively few studies have focused on predicting the therapeutic effect of initial RAI in intermediate- and high-risk DTC patients by detecting pretreatment levels and types of serum cytokines. Therefore, early prediction of response before the initial RAI treatment may have clinical significance for patients with intermediate- and high-risk DTC. Such early prediction can help clinicians identify patients with a higher probability of poor treatment response, optimize the intensity of follow-up, arrange earlier imaging monitoring when necessary, provide personalized risk counseling, and consider additional treatment options. The current study aims to explore whether the types and levels of serum cytokines before initial RAI can be used to predict the therapeutic effect of initial RAI in intermediate- and high-risk DTC patients.

## 2 Materials and methods

### 2.1 Patients

**Figure 1:**
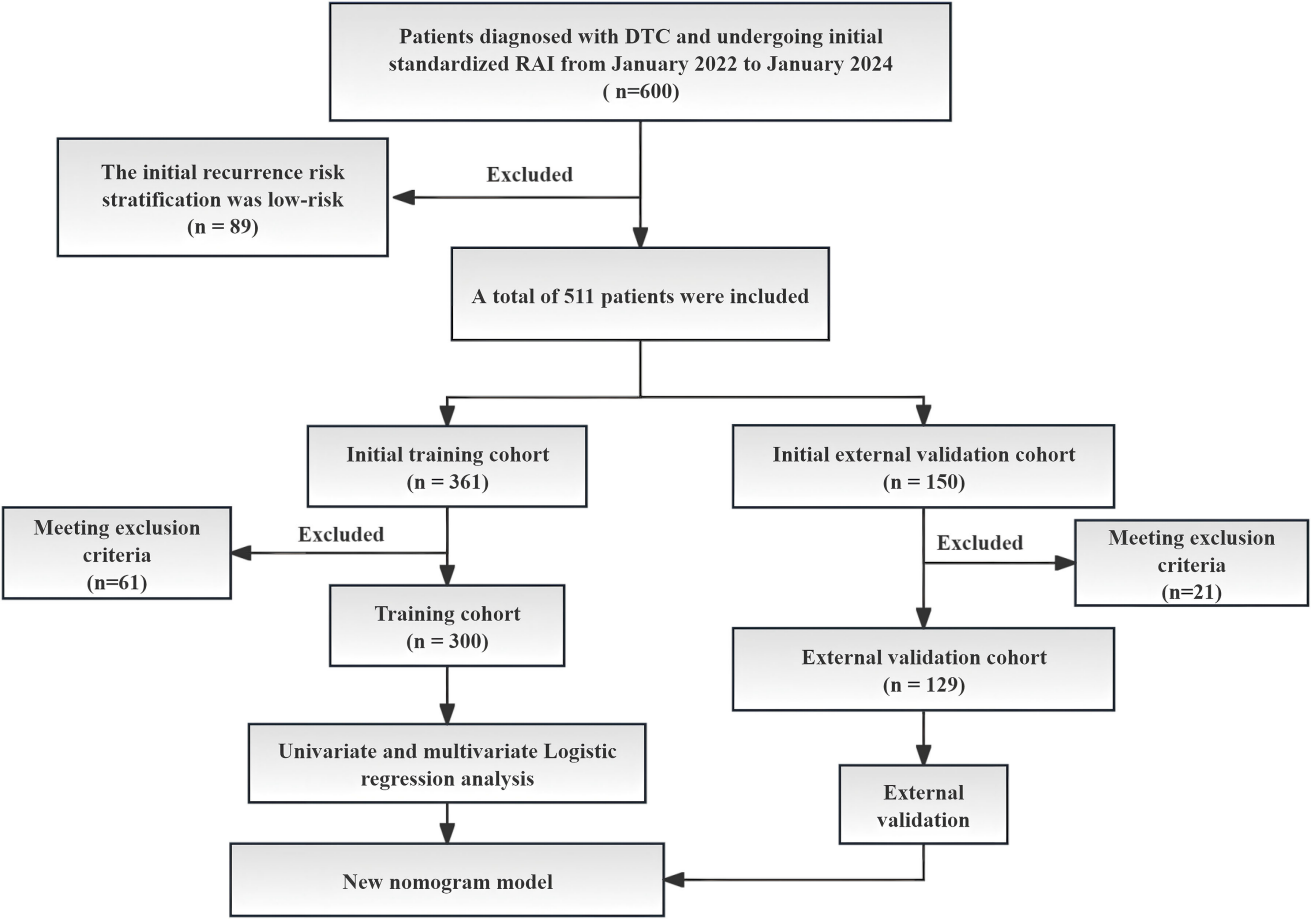
Flowchart for screening patients with intermediate- and high-risk differentiated thyroid cancer and research roadmap. DTC, differentiated thyroid cancer; RAI, radioiodine therapy.

This retrospective analysis was conducted at the Affiliated Hospital of Qingdao University. The study was approved by the Ethics Committee of the Affiliated Hospital of Qingdao University, with a waiver of written informed consent for certain patients (Ethics Approval Number: QYFYWZLL29597). This study was carried out in compliance with the ethical guidelines stipulated in the 1964 Declaration of Helsinki and its subsequent amendments. Patients were retrospectively and consecutively screened from the institutional databases of the two participating centers between January 2022 and January 2024 (**Figure 1**). Initial recurrence risk was classified according to the 2025 ATA risk stratification system. Intermediate-risk disease included patients with microscopic extrathyroidal extension, clinically significant cervical lymph node metastasis, aggressive histological features, vascular invasion, or other ATA-defined intermediate-risk features. According to the ATA risk stratification system, high-risk disease includes patients with gross extrathyroidal extension, incomplete tumor resection, distant metastasis, or other ATA-defined high-risk features. Only patients classified as intermediate- or high-risk non-distant metastatic DTC were eligible for inclusion in the present study.

The inclusion criteria were as follows: (1) total thyroidectomy and selective cervical lymph node dissection; (2) confirmed histopathological diagnosis of DTC; (3) intermediate- or high-risk non-distant metastatic DTC according to the 2025 ATA risk stratification system (12); and (4) initial standardized RAI and serum cytokine testing between January 2022 and January 2024.

The exclusion criteria were as follows: (1) patients diagnosed with inflammation or concomitant inflammatory diseases on the basis of blood tests (e.g., C-reactive protein [CRP] level > 5 mg/L or white blood cell [WBC] count > 9.50×10*9/L) within the past week or other clinical evaluations; (2) patients with concurrent immune system disorders or other malignancies; (3) patients who had recently utilized immunosuppressants or glucocorticoids; (4) patients with immune deficiency caused by non-tumor diseases; (5) patients with positive anti-thyroglobulin antibody (TgAb) test results (a TgAb measurement value < 40.00 IU/ml was considered negative) (13); or (6) patients treated with other antitumor regimens. All patients provided informed consent before treatment.

The training cohort was derived from the Affiliated Hospital of Qingdao University (n = 300), whereas the external validation cohort was obtained from Qingdao Central Hospital, University of Health and Rehabilitation Sciences (n = 129).

### 2.2 Clinical data

This study examined the following clinical data: cytokine levels (IL-2, IL-4, IL-5, IL-6, IL-8, IL-10, IL-12p70, IL-17, TNF-α, IFN-α, and IFN-γ), sex, age, maximum tumor diameter, soft tissue infiltration, BRAF^V600E^ mutation status, lymph node metastasis, stimulated thyroglobulin (sTg), and initial RAI dosage.

### 2.3 Laboratory tests

(1) Detection of blood cytokines: The levels of 11 blood cytokines were determined using a multiplex microsphere flow cytometric immunoassay (Beckman Coulter, Navios flow cytometer, Germany). The normal reference ranges were as follows: (i) IL-2: 0–7.50 pg/mL; (ii) IL-4: 0–8.56 pg/mL; (iii) IL-5: 0–3.10 pg/mL; (iv) IL-6: 0–5.40 pg/mL; (v) IL-8: 0–20.60 pg/mL; (vi) IL-10: 0–12.90 pg/mL; (vii) IL-12p70: 0–3.40 pg/mL; (viii) IL-17: 0–21.40 pg/mL; (ix) TNF-α: 0–16.50 pg/mL; (x) IFN-α: 0–8.50 pg/mL; (xi) IFN-γ: 0–8.50 pg/mL.

(2) Detection of thyroglobulin and antibodies: The levels of sTg and TgAb in patients were determined using an electrochemiluminescence immunoassay (Roche, E170 fully automatic electrochemiluminescence immunoassay analyzer, Switzerland). The normal reference values were as follows: 3.50–77.00 ng/ml and 0–115.00 IU/ml (8, 12, 14). A TgAb measurement value < 40.00 IU/ml was considered negative (13).

### 2.4 RAI protocol and grouping

In accordance with the 2025 ATA guidelines (4), all patients underwent total thyroidectomy. All patients adhered to a low-iodine diet for at least two weeks before their initial RAI and discontinued thyroid hormone for 2–4 weeks to increase endogenous thyroid stimulating hormone (TSH) to > 30 mIU/L. All patients underwent routine testing of sTg and TgAb levels before RAI and completed examinations such as neck thyroid ultrasound, thyroid static imaging, chest CT, and diagnostic ^131^I whole-body imaging/scanning (Dx-WBS). One day before the initial RAI, serum cytokine testing was also conducted. RAI was subsequently carried out. The activity dose of radioactive iodine (RAI) administered was selected based on the risk-adapted strategy outlined in the 2025 American Thyroid Association (ATA) framework. All patients received a conventional adjuvant treatment dose of 100 to 150 millicuries. All patients underwent therapeutic ^131^I whole-body imaging/scanning (Rx-WBS) and SPECT/CT tomographic image fusion within 3 to 7 days after the initial treatment. In accordance with the 2025 ATA guidelines, all patients received regular TSH suppression therapy after initial RAI, and the efficacy of this treatment was evaluated 6 to 12 months later on the basis of serological and imaging examinations.

### 2.5 Definitions of therapeutic response to initial RAI

In this study, patients were divided into four groups on the basis of post-RAI efficacy evaluation: (1) the excellent response (ER) group, in which patients had a suppressed Tg < 0.20 ng/ml or sTg < 1.00 ng/ml with negative TgAb and negative imaging results; (2) the indeterminate response (IDR) group, in which patients had a suppressed Tg < 1.00 ng/ml or sTg < 10.00 ng/ml with negative TgAb and negative imaging results; (3) the biochemical incomplete response (BIR) group, in which patients had a suppressed Tg > 1.00 ng/ml or sTg > 10.00 ng/ml with negative TgAb or increased TgAb levels and negative imaging results; and (4) the structural incomplete response (SIR) group, in which patients had structural or functional evidence of disease with any level of Tg and TgAb levels (4, 15). The latter three groups were combined into the non-ER group.

### 2.6 Statistical analysis

In the training cohort, continuous variables that did not conform to a normal distribution are presented as the median (Q1, Q3), and the Mann–Whitney U test was employed for comparison. Categorical variables are presented as frequencies and/or percentages, and the chi-square test was used for comparisons. Univariate analysis was subsequently carried out. Factors that were significant in the univariate analysis were then incorporated into the multivariate logistic regression model. The predictive and discriminative performance of the model was evaluated using Harrell’s concordance index (C-index), which was used to construct a nomogram model based on the training cohort. Thereafter, in both the training and validation cohorts, the predictive ability of the nomogram was compared and demonstrated by plotting the receiver operating characteristic (ROC) curves of the two cohorts. The calibration curves were subsequently generated using the rms package in the R programming language to mirror the relationship between the predicted treatment outcomes and the actual treatment outcomes. The clinical application value of the nomogram model was assessed using decision curve analysis (DCA) curves, revealing the net benefits for observers. The nomogram was developed using R software (version 4.5.1; https://www.r-project.org/), and all the statistical analyses were carried out using SPSS 29.0 (SPSS Inc., Chicago, IL, USA). The threshold for statistical significance was P < 0.05.

## 3 Results

### 3.1 Clinical characteristics

As illustrated in **Figure 1**, a total of 600 postoperative DTC patients were initially screened between January 2022 and January 2024. A total of 429 patients with intermediate- or high-risk DTC who underwent serum cytokine testing one day prior to their initial RAI were enrolled in this study. Among them, 300 patients from the Affiliated Hospital of Qingdao University constituted the training cohort, whereas 129 patients from Qingdao Central Hospital, University of Health and Rehabilitation Sciences formed the validation cohort. The main clinical features of the patients are summarized in **Table 1**. There were no statistically significant differences in the clinical characteristics listed in **Table 1** between the training cohort and the validation cohort.

**Table 1:** Clinical characteristics of 429 intermediate- and high-risk DTC patients before initial RAI.

Characteristics	Patients n (%)		*t/χ^2^/U*	*P* value
	Training cohort (n = 300)	Validation cohort (n = 129)		
Histological subtype			0.005	0.945^a^
Papillary thyroid carcinoma (PTC)	291 (97.0)	125 (96.9)		
Follicular thyroid carcinoma (FTC)	9 (3.0)	4 (3.1)		
Age (years)			0.474	0.491^a^
< 55	234 (78.0)	99 (76.7)		
≥ 55	66 (22.0)	30 (23.3)		
Gender			0.380	0.538^a^
Male	119 (39.7)	56 (43.4)		
Female	181 (60.3)	73 (56.6)		
Soft-tissue infiltration			0.180	0.671^a^
Yes	208 (69.3)	92 (71.3)		
No	92 (30.7)	37 (28.7)		
BRAF^V600E^ mutation			0.064	0.841^a^
Yes	154 (51.3)	65 (50.4)		
No/NA	146 (48.7)	64 (49.6)		
T stage			0.530	0.467^a^
T1	136 (45.3)	61 (47.3)		
T2	24 (8.0)	18 (14.0)		
T3	58 (19.3)	30 (23.2)		
T4	82 (27.3)	20 (15.5)		
N stage			0.532	0.470^a^
N0	18 (6.0)	5 (3.9)		
N1	282 (94.0)	124 (96.1)		
ATA risk			0.066	0.798^a^
Intermediate risk	258 (86.0)	109 (84.5)		
High risk	42 (14.0)	20 (15.5)		
sTg (ng/mL)	7.64 (3.16, 26.47)	9.34 (4.14, 30.00)	21556.5	0.061^b^
The maximum diameter of the tumor (cm)	1.4 (0.9, 2.1)	1.3 (0.8, 2.0)	18136.5	0.302^b^

Data are expressed as the median (IQR) or frequency. DTC, differentiated thyroid cancer; PTC, papillary thyroid carcinoma; FTC, follicular thyroid carcinoma; RAI, radioiodine therapy; NA, not applicable; T, tumor; N, lymph node; ATA, the American Thyroid Association; sTg, stimulated thyroglobulin before initial RAI; a indicates that the chi-square test was performed; b indicates that the Mann–Whitney U test was performed.

### 3.2 Therapeutic response to initial RAI in 429 intermediate- and high-risk DTC patients

At the last follow-up, the distribution of therapeutic responses in the training cohort was as follows: excellent response (ER), 38.3% (115/300); indeterminate response (IDR), 26.7% (80/300); biochemical incomplete response (BIR), 20.0% (60/300); and structural incomplete response (SIR), 15.0% (45/300). Consequently, the non-excellent response group (IDR + BIR + SIR) accounted for 61.7% (185/300) of the total cohort. In the validation cohort, the proportions of ER, IDR, BIR, and SIR were similar to those in the training cohort (37.2%, 32.5%, 23.2%, and 7.0%, respectively), and the non-excellent response group accounted for 62.8% (81/129).

### 3.3 Risk factors predicting the efficacy of the initial RAI in the training cohort (n=300)

As presented in **Table 2**, univariate analysis revealed statistically significant differences between the ER group and the non-ER group with respect to gender; N stage; ATA risk; maximum tumor diameter; and sTg, IFN-α, IL-10, IL-4, and IL-12p70 levels following initial RAI (all P < 0.05). Conversely, no statistically significant differences were observed between the two groups in terms of age; BRAF^V600E^ mutation status; histological subtype; soft-tissue infiltration; T stage; or TSH, IL-5, IL-2, IL-6, IFN-γ, IL-8, IL-17, or TNF-α levels (all P > 0.05). The results of logistic regression analysis indicated that sTg (OR = 1.070, P < 0.001) and IFN-α (OR = 0.693, P < 0.001) were independent risk factors for predicting the therapeutic effect after initial RAI in intermediate- and high-risk DTC patients (**Table 3**).

**Table 2:** Univariate analysis of ER in 300 intermediate- and high-risk DTC patients following initial RAI.

Characteristics	n	ER group	Non-ER group	*χ^2^/U*	*P* value
Histological subtype				1.019^a^	0.313
PTC	291	113 (98.3%)	178 (96.2%)		
FTC	9	2 (1.7%)	7 (3.8%)		
Age (years)				0.007^a^	0.931
< 55	234	90 (78.3%)	144 (77.8%)		
≥ 55	66	25 (21.7%)	41 (22.2%)		
Gender				5.449^a^	0.020
Male	119	36 (31.3%)	83 (44.9%)		
Female	181	79 (68.7%)	102 (55.1%)		
BRAF^V600E^ mutation				1.392^a^	0.238
Yes	154	64 (55.7%)	90 (48.6%)		
No/NA	146	51 (44.3%)	95 (51.4%)		
Soft-tissue infiltration				3.007^a^	0.083
Yes	208	73(63.5%)	135(73.0%)		
No	92	42(36.5%)	50(27.0%)		
T stage				2.890^a^	0.409
T1	136	56 (48.7%)	80 (43.2%)		
T2	24	7 (6.1%)	17 (9.2%)		
T3	58	25 (21.7%)	33 (17.8%)		
T4	82	27 (23.5%)	55 (29.8%)		
N stage				4.203^a^	0.040
N0	18	13 (11.3%)	5 (2.7%)		
N1	282	102 (88.7%)	180 (97.3%)		
ATA risk				14.430^a^	<0.001
Intermediate risk	258	110 (95.7%)	148 (80.0%)		
High risk	42	5 (4.3%)	37 (20.0%)		
The maximum diameter of the tumor (cm)		1.2 (0.8, 1.9)	1.5 (1.0, 2.5)	8841.0^b^	0.014
sTg (ng/mL)		3.08 (2.19, 5.48)	14.5 (6.59, 52.10)	3212.0^b^	<0.001
IL-5 (pg/mL)		2.21 (1.34, 3.84)	2.26 (1.34, 3.76)	10523.5^b^	0.876
IFN-α (pg/mL)		1.89 (1.48, 4.25)	1.38 (0.90, 2.21)	5846.0^b^	<0.001
IL-2 (pg/mL)		1.83 (1.22, 3.89)	1.87 (1.22, 4.02)	9840.0^b^	0.273
IL-6 (pg/mL)		1.81 (1.17, 3.41)	1.89 (1.17, 3.71)	10364.0^b^	0.706
IL-10 (pg/mL)		1.36 (0.99, 2.04)	1.12 (0.87, 1.97)	8923.0^b^	0.018
IFN-γ (pg/mL)		1.78 (1.73, 4.26)	1.97 (1.73, 4.08)	10595.0^b^	0.953
IL-8 (pg/mL)		1.80 (1.10, 16.23)	2.02 (1.10, 10.25)	9744.0^b^	0.209
IL-17 (pg/mL)		1.85 (1.63, 8.29)	1.85 (1.63, 4.30)	9899.5^b^	0.302
IL-4 (pg/mL)		0.99 (0.66, 1.43)	0.79 (0.66, 1.41)	9057.5^b^	0.029
IL-12p70 (pg/mL)		1.30 (1.21, 1.52)	1.30 (0.89, 1.39)	8968.0^b^	0.019
TNF-α (pg/mL)		0.54 (0.48, 2.03)	1.23 (0.48, 2.03)	9965.5^b^	0.344

DTC, differentiated thyroid cancer; PTC, papillary thyroid carcinoma; FTC, follicular thyroid carcinoma; ER, excellent response; RAI, radioiodine therapy; NA, not applicable; T, tumor; N, lymph node; ATA, the American Thyroid Association; sTg, stimulated thyroglobulin before initial RAI; a indicates that the chi-square test was performed; b indicates that the Mann–Whitney U test was performed.

**Table 3:** Multivariate logistic analysis of ER in 300 intermediate- and high-risk DTC patients after initial RAI.

Characteristics	*P* value	OR	95%CI
Gender (Male/Female)	0.248	1.417	0.785-2.558
N stage (N0/N1)	0.143	2.263	0.758-6.756
ATA risk (Intermediate risk/High risk)	0.095	2.935	0.830-10.380
The maximum diameter of the tumor (cm)	0.693	1.058	0.801-1.397
IFN-α (pg/mL)	<0.001	1.446	1.174-1.780
sTg (ng/mL)	<0.001	0.938	0.912-0.966
IL-10 (pg/mL)	0.138	0.812	0.617-1.069
IL-4 (pg/mL)	0.828	0.938	0.525-1.675
IL-12p70 (pg/mL)	0.790	1.066	0.667-1.703

DTC, differentiated thyroid cancer; ER, excellent response; RAI, radioiodine therapy; N, lymph node; sTg, stimulated thyroglobulin before initial RAI; OR, odds ratio; CI, confidence interval.

### 3.4 Nomogram model for predicting the efficacy of the initial RAI in intermediate- and high-risk DTC patients

**Figure 2:**
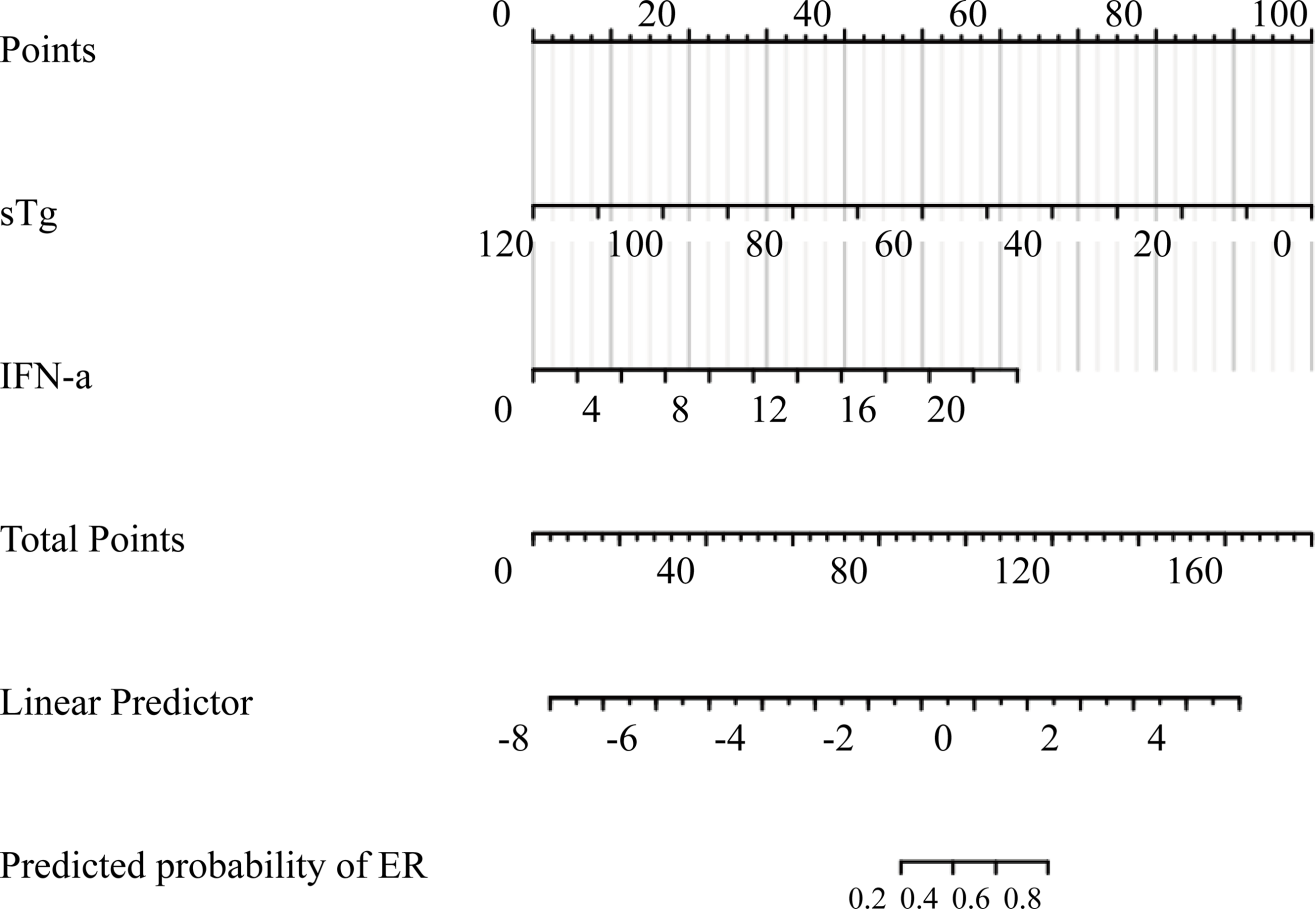
Nomogram for predicting the efficacy of initial RAI in DTC patients with intermediate- or high-risk of recurrence. DTC, differentiated thyroid cancer; RAI, radioiodine therapy; sTg, stimulated thyroglobulin before initial RAI; IFN-α, the level of serum IFN-α before initial RAI; ER, excellent response. Interpretation Guide: For an individual patient: 1) Locate the value of each variable (sTg and IFN-α) on its respective variable axis. 2) For each variable, draw a vertical line upwards to the “score” axis at the top and read the score contributed by that variable. 3) Sum the scores of all the variables to obtain the “total score”. 4) Locate this “total score” value on the “total score” axis at the bottom. 5) Draw a vertical line downwards from the “total score” to the “risk” axis and read the predicted probability. Example: A DTC patient with a medium risk of recurrence has an sTg level of 60 ng/mL (50 points) and an IFN-α level of 14 pg/mL (40 points), with a total score of 90. Drawing a line from 90 on the “total score” axis downwards, the predicted probability of ER efficacy is approximately 26.6%.

A nomogram model was developed on basis of the independent predictors identified through multivariate logistic regression to estimate the probability of an excellent response following initial RAI in intermediate- and high-risk DTC patients. Each variable was assigned a weighted score, and the total score was calculated by summing the scores of all included variables. A vertical line drawn from the total score to the probability axis provided the predicted likelihood of an excellent response. As illustrated in **Figure 2**, sTg had the greatest influence on the prediction, followed by IFN-α.

### 3.5 Comparative validation of the nomogram model

**Figure 3:**
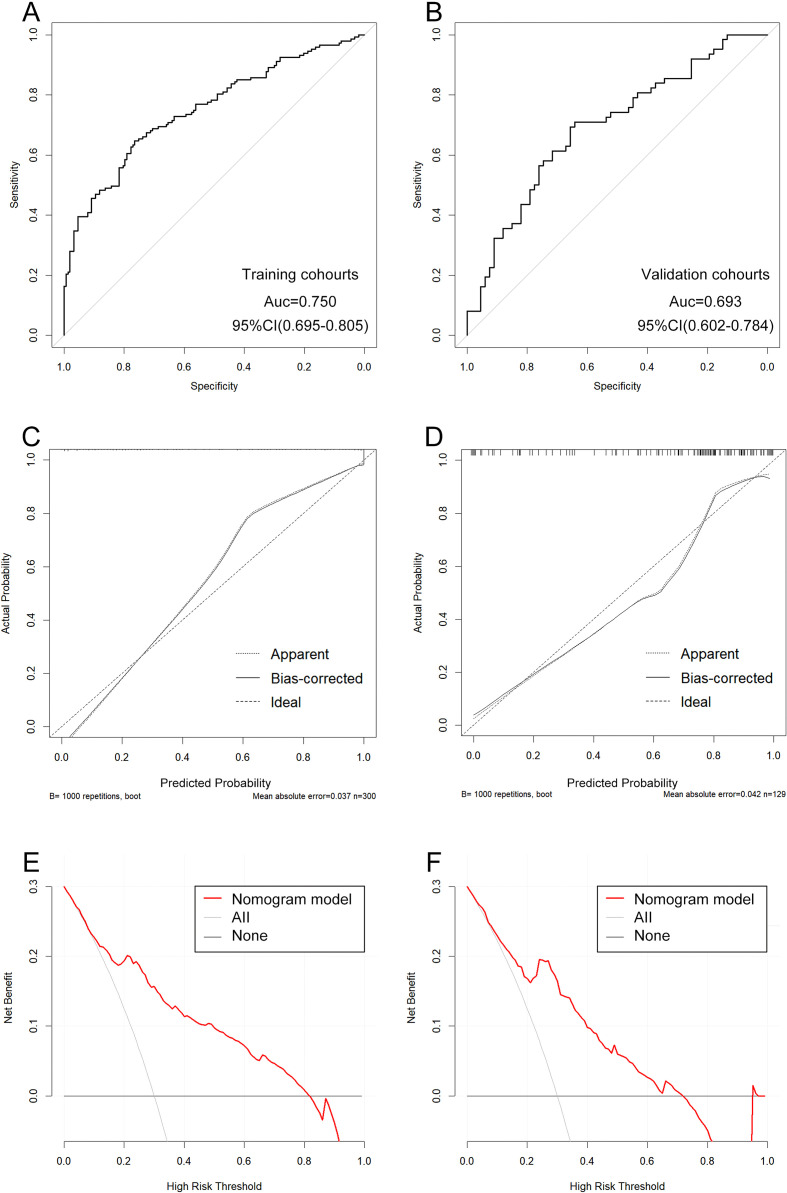
**(A)** ROC curve of the nomogram model for the efficacy of initial RAI in DTC patients with intermediate- or high-risk in the training cohort. **(B)** ROC curve of the nomogram model for the efficacy of initial RAI in DTC patients with intermediate- or high-risk in the validation cohort. **(C)** Calibration curve of the nomogram for predicting the efficacy of initial RAI in DTC patients with intermediate- or high-risk in the training cohort. **(D)** Calibration curve of the nomogram for predicting the efficacy of initial RAI in DTC patients with intermediate- or high-risk in the validation cohort. **(E)** Decision curve analysis of the nomogram for predicting the efficacy of initial RAI in DTC patients with intermediate- or high-risk in the training cohort. **(F)** Decision curve analysis of the nomogram for predicting the efficacy of initial RAI in DTC patients with intermediate- or high-risk in the validation cohort. DTC, differentiated thyroid cancer; RAI, radioiodine therapy; sTg, stimulated thyroglobulin before initial RAI; IFN-α, the level of serum IFN-α before initial RAI.

The nomogram demonstrated strong predictive performance for the efficacy of the initial RAI in intermediate- and high-risk DTC patients. The C-index was 0.750 (95% confidence interval: 0.695–0.805) in the training cohort (**Figure 3A**) and 0.693 (95% confidence interval: 0.602–0.784) in the validation cohort (**Figure 3B**). The calibration curves indicated good agreement between the predicted and observed probabilities of an excellent response in both cohorts (**Figures 3C, D**). ROC analysis yielded an AUC of 0.750 in the training cohort (**Figure 3A**) and 0.693 in the validation cohort (**Figure 3B**). Decision curve analysis further confirmed the clinical utility of the nomogram, demonstrating a favorable net benefit across a range of threshold probabilities (**Figures 3E, F**).

## 4 Discussion

Thyroid cancer is one of the most common endocrine malignancies in clinical practice, and DTC accounts for more than 90% of all thyroid cancer cases (1, 2). DTC has a relatively good prognosis. Recent research indicates that its 5-year overall survival rate is approximately 98% (3). The 2025 guidelines of the American Thyroid Association (ATA) suggest that the standard treatment protocols for DTC include surgical resection, thyroid hormone suppression therapy, and RAI (3, 4). RAI is an important adjuvant treatment after thyroid cancer surgery; it is commonly used to eliminate residual thyroid cancer tissues and microscopic metastatic foci in patients with moderate and high recurrence risk after surgery, thereby reducing the recurrence risk of DTC and improving the prognosis. Previous studies have shown that cytokines also play important roles in regulating the tumor microenvironment and radiosensitivity (9–11). In this retrospective analysis, the training cohort included 300 patients who underwent initial RAI treatment in the Department of Nuclear Medicine of the Affiliated Hospital of Qingdao University. The findings of this study, verified by an external validation cohort, indicated that the sTg level and the IFN-αlevel prior to initial RAI treatment were indeed independent risk factors for the therapeutic outcome of RAI treatment. Consequently, in this study, we developed a novel quantitative nomogram model based on a training cohort to predict the efficacy of initial RAI treatment in patients with intermediate- to high-risk DTC.

Tg is a large, 660-kDa molecular glycoprotein complex secreted by thyroid follicular epithelial cells. After being synthesized within thyroid cells, it is secreted into the follicular lumen for storage. Tg is one of the main secretions of thyroid follicular cells and an important precursor of thyroid hormones. Tumor cells derived from well-differentiated thyroid follicles retain the ability to secrete Tg (16). The serum level of Tg can, to a certain extent, reflect the tumor burden. Currently, the serum Tg level before treatment is often detected in clinical practice as an important biomarker for evaluating the therapeutic effect of RAI in DTC patients and monitoring the risk of tumor recurrence. The study by Park et al. (17) reported that sTg is an independent risk factor for the therapeutic effect of initial RAI in DTC patients. When the sTg level is below 10 ng/mL, there is a high likelihood of a satisfactory therapeutic effect; conversely, a higher sTg level suggests a poor therapeutic effect or the possibility of tumor recurrence, requiring further intervention or adjustment of the treatment plan. This study also revealed that sTg is an independent risk factor for the therapeutic effect of initial RAI in intermediate- and high-risk DTC patients, which is consistent with the results of the aforementioned study. This finding further validates the utility of using sTg as a reliable biomarker for predicting the efficacy of initial RAI treatment in our study.

Cytokines are crucial regulatory factors of the immune system that are capable of modulating the tumor microenvironment and radiosensitivity (9–11). They can also influence the uptake and transport of radioactive iodine by regulating the activity of immune cells within the tumor microenvironment. A previous *in vitro* basic study by Misaki et al. (18) has verified this finding. Previously, Yang et al. (19) reported that the serum IL-2R level after RAI could be used to assess the therapeutic effect of ^131^I in DTC patients, whereas Jiang et al. (20) found that the expression levels of miR-221 and IL-17 in PTC were significantly elevated and positively correlated. In this study, we conducted a retrospective investigation on the predictive value of 11 serum cytokines for initial RAI response in the human body. The results demonstrated that IFN-α was an independent risk factor influencing the therapeutic effect of initial RAI in DTC patients. In intermediate- and high-risk DTC patients with relatively high serum IFN-α levels, the ER rate after RAI was significantly increased (OR = 1.446, P < 0.001). This is in full accordance with the perspective presented by the nomogram model developed in this study. This is the first time that the serum IFN-α level has been utilized to predict the therapeutic effect of initial RAI in intermediate- and high-risk DTC patients.

As a member of the type I interferon family, IFN-α has dual antitumor and immunoregulatory functions. It can activate the JAK-STAT signaling pathway and upregulate the expression of MHC class I molecules. This facilitates the recognition and killing of tumor cells by CD8+ T cells, reducing the tumor burden and increasing the sensitivity of residual lesions to RAI. Furthermore, IFN-α can enhance the antitumor immune response by activating dendritic cells and CD8+ T cells and regulating other related cytokines, such as IFN-γ (18), in combination with the cytotoxic effect of RAI. Previous studies have indicated that the activities of CD4+ T cells, the CD4/CD8 ratio, and the level of NK cells in patients with thyroid cancer are significantly lower than those in normal individuals, whereas the increased activity of CD8+ T cells may be related to the activation effect of IFN-α on CD8+ T cells (8), which is consistent with the results of the present study. The abovementioned perspective is also corroborated by the validation of the nomogram model developed in this study using the external validation cohort.

Notably, the univariate analysis in this study revealed that several other cytokines, including interleukin-4 (IL-4) and interleukin-12p70, were also significantly associated with the efficacy of the RAI treatment. However, in the multivariate logistic model incorporating IFN-α and other relevant factors, they failed to retain their independent predictive power. As previously described, this phenomenon can likely be ascribed mainly to differences in the number of enrolled patients and their baseline characteristics (18–20). Nonetheless, the significance of IL-4 and IL-12p70 in the univariate analysis still underscores their potential biological implications. The loss of their predictive ability does not categorically negate their biological relevance. Future research should employ larger sample sizes or more targeted experimental designs to further investigate the intricate interactions among these factors and their individual contributions.

This study initially discussed the predictive value of serum IFN-α levels for the efficacy of initial RAI treatment; however, several limitations remain: (1) This study was a two-center retrospective study with a relatively limited sample size (n = 429). Moreover, the sample inclusion and exclusion procedures in this study might have been subject to inevitable selection biases. (2) The occurrence and development of thyroid cancer are closely related to the activation of proto-oncogenes, and cytokines can regulate the expression of proto-oncogenes. Gene mutations such as BRAF^V600E^ are relatively common genetic variations in thyroid cancer. This study did not explore the influence of related gene mutations on the predictive value of serum cytokine levels. (3) IFN-α, an important component of the cytokine family of interferons, is susceptible to other factors, such as infections or autoimmune diseases. In this study, only samples affected by such factors were excluded according to the inclusion and exclusion criteria. However, the impact of related factors, such as CRP and WBC levels, on IFN-α was not explored. (4) Furthermore, to maintain model interpretability and avoid potential multicollinearity among closely related disease-burden variables, the present nomogram did not simultaneously incorporate all detailed clinicopathological characteristics associated with ATA recurrence risk stratification. Although this strategy improved model simplicity and clinical applicability, the potential incremental contribution of additional pathological features warrants further investigation in future studies.

## 5 Conclusion

The sTg and serum IFN-α levels of intermediate- and high-risk DTC patients before initial RAI have predictive value for therapeutic efficacy. Through systematic research and validation, this study developed a novel quantitative nomogram model. This model can visually estimate the efficacy of initial RAI in patients with intermediate- and high-risk DTC. This study provides additional support for individualized clinical decision-making and the refinement of patient management strategies. The model may help estimate response probability and support risk stratification, pending further prospective validation before routine clinical application.

## Data Availability

The raw data supporting the conclusions of this article will be made available by the authors, without undue reservation.
